# Amide proton transfer-weighted imaging combined with multiple models diffusion-weighted imaging of endometrial cancer: correlations between multi-modal MRI parameters and HIF-1α expression

**DOI:** 10.3389/fonc.2025.1556311

**Published:** 2025-04-28

**Authors:** Jun Li, Changjun Ma, Shifeng Tian, Ailian Liu, Qingling Song, Nan Wang, Qingwei Song, Liangjie Lin, Peng Sun, Jiazheng Wang

**Affiliations:** ^1^ Department of Radiology, The First Affiliated Hospital of Dalian Medical University, Dalian, Liaoning, China; ^2^ School of Biomedical Engineering, Faculty of Medicine, Dalian University of Technology, Dalian, Liaoning, China; ^3^ Dalian Medical Image Artificial Intelligence Engineering Technology Research Center, Dalian, Liaoning, China; ^4^ Technology Innovation Center of Hyperpolarized MRI, Dalian, Liaoning, China; ^5^ Philips Health Technology (China) Co., Ltd., Beijing, China

**Keywords:** DWI, IVIM, HIF-1α, endometrial cancer, amide proton transfer weighted imaging

## Abstract

**Background:**

Hypoxia inducible factor (HIF-1α) is a major transcriptional factor regulating gene expression under hypoxic conditions. HIF-1α expression was closely correlated with the oxygenation status of tumor and could serve as an important biomarker for tumor hypoxia, aggressiveness, or radiation resistance. High expression of HIF-1α contributes to high aggressiveness or poor prognosis of endometrial cancer.

**Purpose:**

This study aimed to investigate correlations between multimodal MRI parameters (derived from amide proton transfer weighted imaging [APTw], conventional diffusion weighted imaging [DWI], intravoxel incoherent motion [IVIM] imaging and diffusion kurtosis imaging [DKI]) and HIF-1α expression, and to determine whether multimodal MRI can be used for quantitative evaluation of HIF-1α expression.

**Study type:**

Retrospective.

**Population:**

A total of 94 patients with EC were examined with 32 cases finally included in the high HIF-1α expression group and 40 cases included in the low expression group according to the exclusion and inclusion criteria.

**Field Strength/Sequence:**

3.0T/APTw, DWI, IVIM, and DKI

**Assessment:**

The asymmetry of magnetization transfer rate (MTR_asym_), apparent diffusion coefficient (ADC), pure diffusion coefficient (D), pseudo diffusion coefficient (D*), perfusion fraction (f), mean kurtosis (MK), and mean diffusivity (MD) were calculated from multimodal MRI and compared between HIF-1α high expression and HIF-1α low expression groups.

**Statistical Test:**

Mann–Whitney U-test; Chi-square test or Fisher exact test; logistic regression analysis; Area under the receiver operating characteristic (ROC) curve (AUC); The Delong test; Pearson or Spearman correlation coefficients. The significance threshold was set at *P* < 0.05.

**Result:**

MTR_asym_, ADC, D, D*, MK and MD values were significantly higher in high HIF-1α expression than in low HIF-1α expression groups, whereas f value was significantly lower in high HIF-1α expression than in low HIF-1α expression groups. The AUC of HIF-1 α expression evaluated by MTR_asym_, ADC, D, D*, f, MD, MK and their combination were 0.894 (0.740, 0.973), 0.746 (0.568, 0.879), 0.716 (0.528, 0.904), 0.920 (0.772, 0.984), 0.756 (0.578, 0.886), and 0.973 (0.851-1.000), respectively. Multivariate analysis revealed that only f, MK, and MD values were independent predictors for evaluating HIF-1α expression in EC.

**Conclusion:**

APTw combined with multi-model diffusion imaging can quantitatively evaluate the expression of HIF-1α in EC, and the combination of multiple quantitative parameters can improve the evaluation efficiency.

## Introduction

Endometrial cancer (EC) is a neoplasm that arises from the endometrium and is the second most prevalent malignancy affecting the female reproductive system in China (1, 2). It is the most frequently diagnosed gynecological tumor in developed nations, with a steadily increasing incidence (3). Approximately 70% of EC cases are localized to the uterine body, representing an early clinical stage with a favorable prognosis (4). Surgery intervention is the primary treatment for EC, with radiotherapy, chemotherapy, and hormone therapy often used as adjuvant therapies. tumors are divided into four subgroups: polymerase-epsilon (POLE) mut, protein 53 (p53) wild type, p53 missense mutations and mismatch repair deficient (5). In the face of such a complex disease classification, when choosing the treatment of endometrial cancer, we should consider the patient’s age, pathological type, molecular classification and clinical stage (low, medium, high risk) and other factors, in order to optimize the outcome of patients (6). Typically, patients with stage I and stage II endometrial cancer with localized tumors opt for hysterectomy in the absence of high-risk factors. Conversely, patients with high-risk factors, such as extrauterine metastasis, may benefit from concurrent radiotherapy and chemotherapy to enhance treatment efficacy (7, 8). Progesterone therapy is typically recommended as the initial treatment for EC patients seeking to preserve their fertility (9). At the same time, systematic assessment, such as microsatellite instability, has been practiced and applied in clinical practice. For patients with this specific biomarker, Programmed Death-1/Programmed Death-Ligand 1 inhibitors have shown promising therapeutic outcomes (10). Radiotherapy plays a crucial role in the treatment of advanced stage EC patients with high-risk features, such as extrauterine invasion, although individual responses to this treatment may vary. Numerous variables influence the efficacy of radiotherapy, including cellular processes such as growth and apoptosis, the presence of a hypoxic microenvironment, angiogenesis, and temperature. Among these factors, hypoxia inducible factor (HIF-1α) is specifically associated with the hypoxic microenvironment (11, 12).

HIF-1α serves as a key transcription factor that regulates gene expression under hypoxia (13–15). Prior studies have shown that (14, 16, 17) the expression of HIF-1α is closely related to the oxygenation state of the tumor. In normoxic conditions, HIF-1α undergoes rapid degradation, whereas in hypoxic environments, the degradation of HIF-1α is inhibited, leading to its accumulation in the nucleus. This phenomenon can serve as a significant biomarker for tumor hypoxia, invasiveness, and resistance to radiation therapy (18). Additionally, HIF-1α plays a crucial role in regulating various cellular functions in response to low oxygen levels, including glucose uptake, energy metabolism, angiogenesis, erythropoiesis, cell proliferation, apoptosis, cell-cell and cell-matrix interactions, which collectively contribute to processes such as tumorigenesis, metastasis, and epithelial-mesenchymal transformation (EMT) (19, 20). Conversely, elevated levels of HIF-1α can impact the efficacy of tumor therapy by modulating downstream and upstream molecular signaling pathways and influencing the expression of hypoxia-related genes involved in angiogenesis, erythropoiesis, glycolysis, cell adhesion, cell proliferation, and apoptosis (21), meanwhile, causing inadequate arterial blood supply, reduced vascular density, impaired vascular tissue transport efficiency, alterations in red blood cell flow, functional shunting, and imbalance of oxygen supply and demand (22). These effects ultimately contribute to tumor hypoxia and heightened invasiveness of endothelial cells, leading to an unfavorable prognosis for patients with EC and impacting treatment outcomes. Generally speaking (23), in the context of tumor hypoxia, the efficacy of tumor radiotherapy and chemotherapy is typically diminished, necessitating the assessment of tumor hypoxia status to inform clinical interventions. Through an understanding of the molecular pathways involving HIF-1α, novel therapeutic approaches targeting highly expressed HIF-1α signaling pathways have been devised to enhance personalized and precise treatment for cancer patients (24, 25).

Previously, the identification of tumor molecules relied on invasive surgical procedures or biopsies to obtain tissue samples. However, the presence of tumor heterogeneity posed a challenge to the efficacy of these samples, as small tissue samples were unable to accurately represent the entire tumor (26). In addition, although impact genomics has been used in molecular**/**genome analysis, it is hindered by the widespread use of factors such as technical complexity (27, 28). Previous methods for assessing tumor hypoxia included direct approaches such as the use of oxygen sensing probes (29) and phosphorescence lifetime imaging to measure PO_2_ (30), as well as indirect methods like oxygen-enhanced magnetic resonance imaging (OE-MRI) (31), magnetic susceptibility imaging (19), and positron emission tomography (PET) (32) to infer tumor hypoxia. At the same time, some studies have also found that there is a correlation of the quantitative parameters measured by dynamic contract-enhanced magnetic resonance imaging (DCE-MRI) (33), diffusion weighted imaging (DWI) (34) and intravoxel incoherent motion (IVIM) (35) with tumor hypoxia. Among all these imaging parameters, no individual one can serve as a definitive marker for assessing tumor oxygenation. Conversely, the utilization of multiple imaging parameters obtained through multi-parameter imaging technology is anticipated to have a greater impact on the assessment of tumor hypoxia. Thus, this study employed a combination of amide proton transfer weighted imaging (APTw), DWI, IVIM, and diffusion kurtosis imaging (DKI) to examine the expression of EC HIF-1α in relation to tumor metabolism and blood perfusion.

## Materials and methods

### Study population

**Figure 1:**
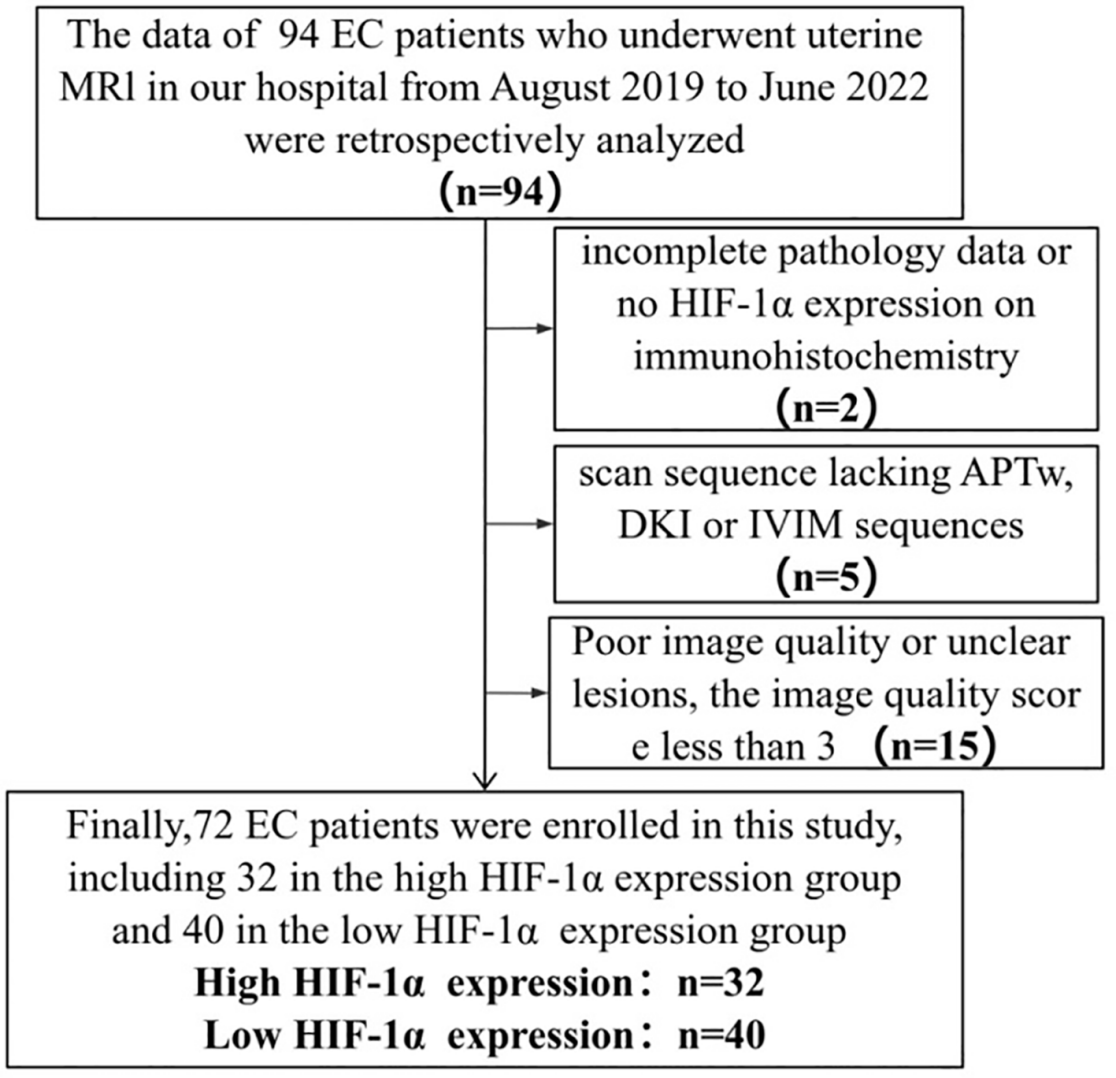
Flow chart of patient enrollment.

The Ethics Committee approved the retrospective study and waived the requirement for informed consent. The retrospective analysis examined the clinical and imaging data of 94 patients who underwent 3.0T MR examination at our hospital between August 2019 and June 2022, and were subsequently diagnosed with EC following uterine curettage or pathology. Inclusion criteria for the study encompassed the presence of high-quality MRI images with clearly delineated lesions devoid of artifacts, facilitating the accurate identification of tumor boundaries during region of interest (ROI) delineation. Additionally, the inclusion criteria stipulated the presence of a solitary tumor without concurrent tumors or endometrial hyperplasia, as well as the absence of prior treatment for endometrial carcinoma prior to MRI examination. Exclusion criteria encompassed the absence of essential scan sequences such as APTw, DWI, IVIM, and DKI, suboptimal MRI image quality resulting in indistinct lesion visualization or tumor size less than 1cm, and incomplete clinicopathological data including the lack of HIF-1α expression information. The flow chart of the incoming and outgoing group is shown in **Figure 1**. Finally, 72 patients were enrolled in this study. According to the expression of HIF-1α, they were divided into two groups: high expression of HIF-1α (n = 32) and low expression of HIF-1α (n = 40). The general clinicopathological data of the two groups of patients were collected through our hospital information management system, including age, differentiation degree, menopausal state, Federation International of Gynecology and Obstetr (FIGO) staging, deep myometrium invasion (DMI), lymph-vascular space invasion (LVSI), lymph node metastasis (LNM), and pathological type, as shown in **Table 1**.

**Table 1:** Patient characteristics.

	n	High HIF-1α expression n=32	Low HIF-1α expression n=40	χ^2^/t	*P*
**Age (y)^1^**	72	59.44 ± 10.15	58.25 ± 9.18	0.520	0.604
FIGO Stage *n (%)*					
**Stage I**	52	24/32 (75.00)	28/40 (70.00)	3.275	0.333
**Stage II**	6	4/32 (12.50)	2/40 (5.00)		
**Stage III**	12	3/32 (9.38)	9/40 (22.50)		
**Stage IV**	2	1/32 (3.12)	1/40 (2.50)		
Differentiation degree *n (%)*					
**Low**	26	15/32 (46.88)	11/40 (27.50)	4.011	0.136
**Medium**	34	11/32 (34.38)	23/40 (57.50)		
**High**	12	6/32 (18.75)	6/40 (15.00)		
Menopausal state *n (%)*					
**Before**	14	7/32 (21.88)	7/40 (17.50)	0.217	0.767
**After**	58	25/32 (78.13)	33/40 (82.50)		
Pathological type *n (%)*					
**Type I**	23	11/32 (34.38)	12/40 (30.00)	0.157	0.801
**Type II**	49	21/32 (65.63)	28/40 (70.00)		
Irregular vaginal bleeding *n (%)*					
**No**	43	15/32 (46.88)	28/40 (70.00)	3.952	***0.040***
**Yes**	29	17/32 (53.12)	12/40 (30.00)		
DMI *n (%)*					
**<1/2 muscular layer**	19	11/32 (34.38)	8/40 (20.00)	1.891	0.189
**≥1/2 muscular layer**	53	21/32 (65.62)	32/40 (80.00)		
LVSI *n (%)*					
**Positive**	14	7/32 (21.88)	7/40 (17.50)	0.217	0.767
**Negative**	58	25/32 (78.12)	33/40 (82.50)		
LNM *n (%)*					
**Positive**	11	3/32 (9.38)	8/40 (20.00)	1.551	0.325
**Negative**	61	29/32 (90.62)	32/40 (80.00)		

HIF-1α, hypoxia inducible factor; FIGO, Federation International of Gynecology and Obstetr; DMI, deep myometrium invasion; LVSI, Lymph-vascular space invasion; LNM, lymph node metastasis.

The bold values in the tables indicate p-values < 0.05, denoting statistical significance.

### MRI technique

MR scans were performed on a 3.0T MR scanner (Ingenia CX, Philips Healthcare, Best, the Netherlands) with a 32-channel abdominal coil. Before the examination, the patient was instructed to empty the bladder, and the intrauterine device was taken out one day before the examination. The patient was in the supine position with the feet advanced. The MRI sequences included transverse T2-weighted imaging (T2WI), sagittal T2WI, diffusion-weighted imaging (DWI) (b=0, 800 s/mm^2^), APTw, DKI (3 b values: 0, 1000, 2000 s/mm^2^ and diffusion gradients were applied in 32 orthogonal directions) and IVIM (10 b values: 0, 20, 50, 100, 150, 200, 400, 800, 1200, 2000 s/mm^2^), and the specific parameters are shown in **Table 2**.

**Table 2:** Main imaging parameters of the MRI sequences.

Series	Orientation	TR/TE (ms)	FOV (mm^3^)	ACQ Voxel (mm^3^)	Thickness/ Gap(mm)	Slices	Scan Time (Min sec)
T_2_WI	TRA	4596/95	240 × 240 × 99	0.7 × 0.7 × 4.0	4.0/1.0	20	1 min 14 s
T_2_WI	SAG	4930/84	250 × 250 × 99	0.95 × 0.95 × 4.0	4.0/1.0	20	2 min 08 s
DWI	COR	7800/72	380 × 380 × 105	3.0 × 3.0 × 3.0	3.0/0	35	3 min 31 s
IVIM	SAG	2500/94	380 × 380 × 65	3.0 × 3.0 × 5.0	5.0/1.0	11	5 min 23 s
DKI	SAG	1997/89	380 × 356 × 95	3.0 × 3.0 × 5.0	5.0/1.0	16	5 min 29 s
APTw	SAG	6416/7.8	130 × 130 × 49	2.0 × 2.0 × 7.0	7.0/0	7	5 min 53 s

TR, repetition time; TE, echo time; FOV, Field of View; ACQ, Acquisition; T2WI, T2-weighted Imaging; DWI, Diffusion-Weighted Imaging; IVIM, Intravoxel Incoherent Motion; DKI, Diffusion Kurtosis Imaging; APTw, Amide Proton Transfer weighting; TRA, Transverse; SAG, Sagittal; COR, Coronal.

IVIM imaging evaluates the diffusion motion component and blood perfusion component separately through modelling of related quantitative parameters on the diffusion weighted images. The relationship between the signal change and all b-values can be expressed by the following equation (36).


$${\text{S}}_{\text{b}}/{\text{S}}_{0}=\;\left(1-\text{f}\right)\;\cdot \text{ exp }\left(-\text{bD}\right)\;+\text{ f }\cdot \text{ exp }\left[-\text{b }\left(\text{D}*+\text{D}\right)\right],$$

where b is the diffusion sensitivity factor, S_0_ and S_b_ represent the signal intensities of b=0 s/mm^2^ and all other b values, respectively. The f value is the perfusion fraction (between 0 and 1), which represents the volume ratio of the microcirculation perfusion in the voxel to the overall diffusion effect; the D value is the pure diffusion coefficient, which represents the pure water molecule diffusion movement motion component; D* is the pseudo diffusion coefficient produced by blood circulation, which represents the incoherent motion of the microcirculation in the voxel; that is, the rapid diffusion motion related to perfusion. The IVIM data were processed on the Intellispace Portal v10.0 workstation (Philips Healthcare) using the advanced diffusion analysis tool.

APTw imaging was performed using a 3-dimensional (3D) turbo-spin-echo sequence with chemical shift-selective fat suppression. The middle slice of APTw images was located through the largest cross-section of the selected tumor lesion present on conventional MR images. Data were acquired with seven saturation-frequency offsets (± 2.7, ± 3.5, ± 4.3, and -1,540 ppm) for fitting of the Z-spectrum. Saturation radio-frequency pulses for APTw imaging were implemented with an amplitude of 2µT and a duration of 2 s. The acquisition was repeated three times at +3.5 ppm with shifted echo times for generation of B0 maps. B0-corrected ATPw images were reconstructed online. The MTR_asym_ (magnetization transfer ratio asymmetry) value at the frequency offset of +3.5 ppm was calculated as percent level (relative to S_0_) for APTw quantitative analysis:


$${\text{MTR}}_{\text{asym}}\left(3.5\text{ ppm}\right)\times \;100\%\;=\;({\text{S}}_{\text{sat}}(-3.5\text{ ppm})/{\text{S}}_{0}-{\text{S}}_{\text{sat}}\left(+3.5\text{ ppm}\right)/{\text{S}}_{0}))\times 100\%$$

where S_0_ is the water signal strength at a saturation frequency of -1540 ppm, and S_sat_ is the water signal strength at a saturation frequency of +3.5/-3.5 ppm after B0 correction.

DKI uses 3 b values (0, 1000, and 2000 s/mm^2)^ and 32 orthogonal directions to obtain DKI parameters of fractional anisotropy (FA), mean kurtosis (MK) and mean diffusivity (MD) by the following equation (37):


$$\text{S}\left(\text{b}\right)=\text{S}\left(0\right)\cdot \text{exp}(-\text{b}\cdot \text{MD}+1/6\cdot {\text{b}}^{2}\cdot {\text{MD}}^{2}\cdot \text{MK})$$

where S(0) is the DWI signal of bounded 0, and S(b) is the DWI signal of a specific b value. MD represents the average diffusion coefficient, reflecting the complexity of the tissue structure, while MK represents the average diffusion kurtosis, reflecting the overall diffusion level and diffusion resistance of water molecules.

### Image analysis

**Figure 2:**
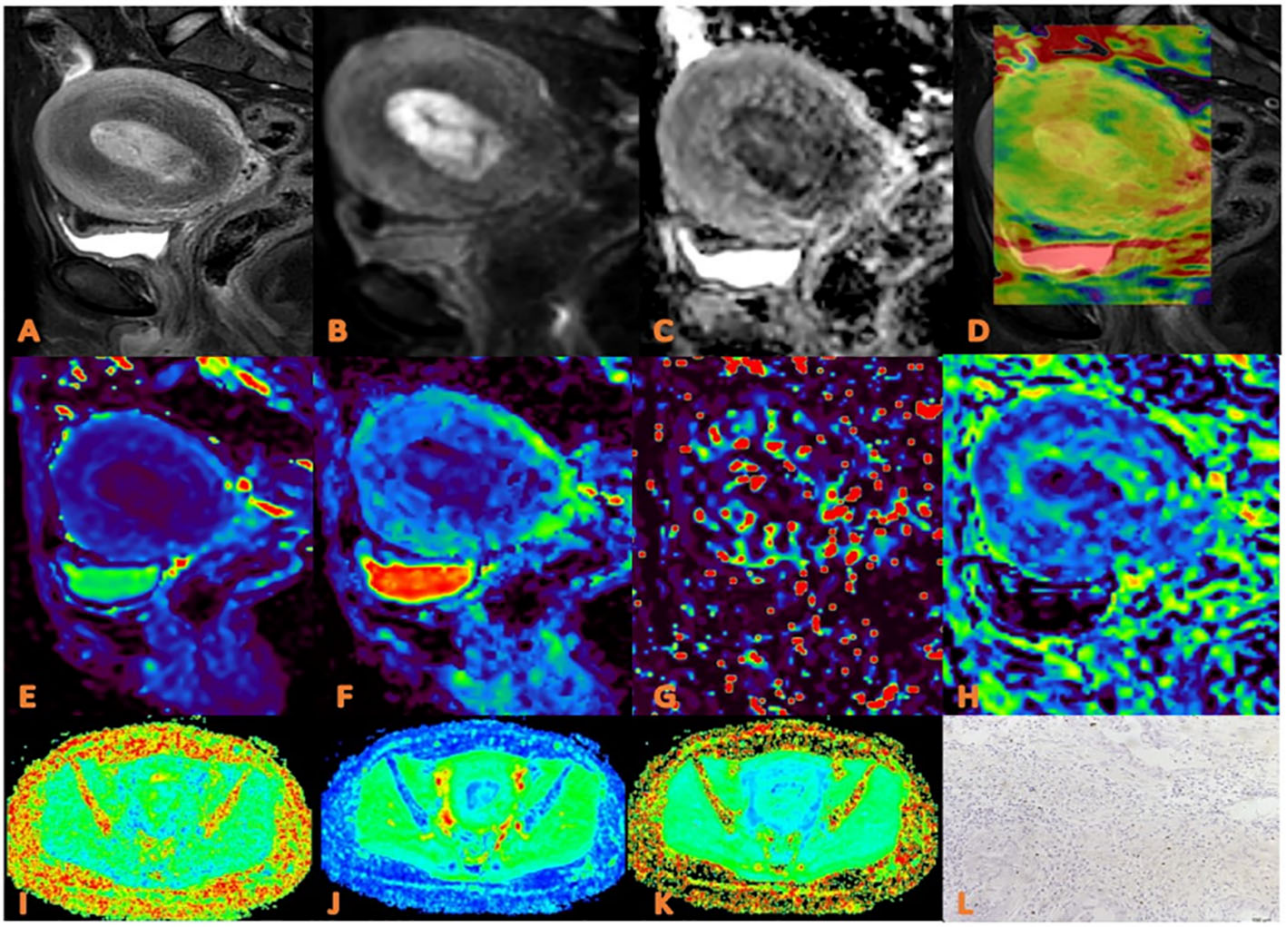
APTw, DKI and IVIM parameters for an EC patient with low HIF-1α expression. **(A)** sagittal T2WI, showing a slightly high signal mass in the uterine cavity; **(B)** sagittal DWI image; **(C)** sagittal ADC image; **(D)** APTw fused with T2WI (mean MTRasym value 2.90%) **(E–H)** ADC, D, D*, and f images. Mean values are 0.750 ×10-3 mm2/sec for ADC, 0.470 × 10-3 mm2/sec for D, 0.560×10-2 mm2 /sec for D* and 0.30% for f; **(I–K)** FA, MK, and MD images. Mean values are 0.314 for FA, 0.555 for MK, and 0.876 μm2/ms for MD; **(L)** Immunohistochemical staining image (×200) showed that HIF-1α expression of the tumor appeared as low expression.

**Figure 3:**
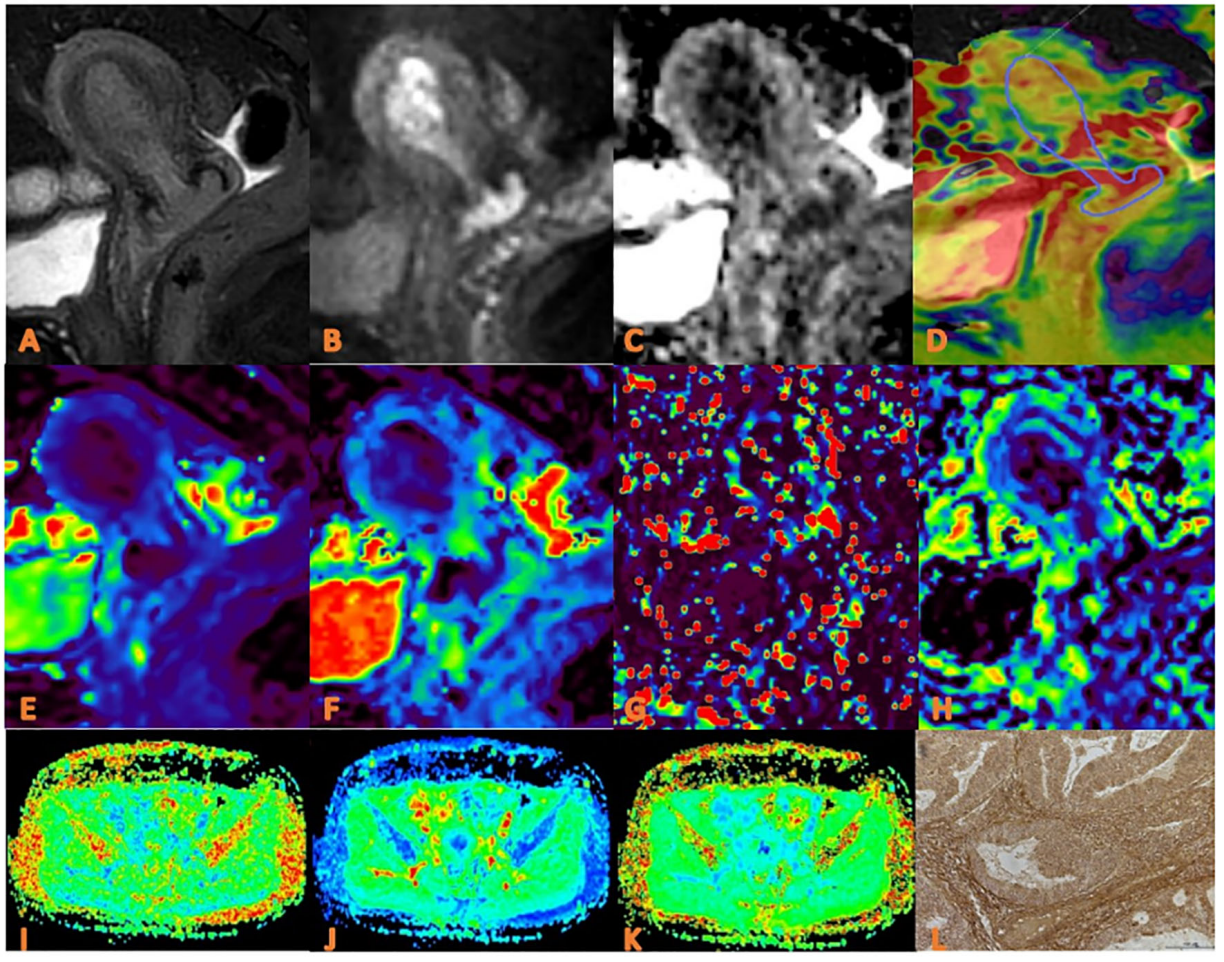
APTw, DKI and IVIM in EC that had high HIF-1α expression. **(A)** sagittal T2WI, showing a slightly high signal mass in the uterine cavity; **(B)** sagittal DWI image; **(C)** sagittal ADC images; **(D)** APTw and T2WI fusion images (mean APT value 3.07%) **(E–H)** ADC, D, D*, and f images. Mean values are 0.760×10^-3^ mm^2^/sec for ADC, 0.590 × 10^-3^ mm^2^/sec for D, 0.600 ×10^-2^ mm^2^/sec for D* and 0.160% for f; **(I–K)** FA, MK, and MD images. Mean values are 0.395 for FA, 0.674 for MK, and 0.866μm^2^ /ms for MD; **(L)** Immuno-histochemical staining image (×200) showed that HIF-1α expression of the tumor appeared as high expression.

Image analysis and data measurement were performed independently by three radiologists (TS F, MC J and LJ, with 10, 4 and 2 years of experience in uterine MR readings, respectively) who were blinded to the clinical and imaging data. The APTw and IVIM images were transferred to Intellispace Portal workstation, and the DKI images were transferred to GE AW4.6 workstation for post-processing. The specific measurement method is as follows: First, the maximum cross-sectional tumor on the conventional T_2_WI and DWI image was located; Second, each parameter map were merged with the DWI images (b=800 s/mm^2^) of the same layer to draw the regions of interest (ROIs) on the maximum cross-sectional tumor by using a freehand tool, which should include as many solid areas of the tumor as possible (**Figures 2**, **3**). Every ROI was carefully positioned to avoid necrosis, hemorrhage, cystic degeneration, blood vessels, and partial volume effects on the edge of tumors. The mean value from the ROI for each parameter was recorded for further analysis.

### Pathologic analysis

The paraffin blocks of endometrial cancer tissues submitted for examination in the pathology department of our hospital were collected retrospectively, and then 4 μm sections were made by a pathology technician with 10 years of pathological wax section experience, and sealed and preserved in a cool and dark environment. Then a graduate student with 3 years’ experience of immunohistochemical experiment performed HIF-1α immunohistochemical experiment in the pathology laboratory, and the staining was observed by two-step immunohistochemical method. The specific staining process included: baking, dewaxing, antigen repair, blocking endogenous peroxidase, first antibody incubation, second antibody incubation, Diaminobenzidine Horseradish Peroxidase Color Development Kit working solution coloration, re-staining and dehydration sealing. The staining results were evaluated by two doctors with 3 and 4 years’ pathology experience respectively without knowing the clinical and imaging information. When results of the two assessments were inconsistent, they discussed and agreed with another pathologist with 10 years of experience. Among them, HIF-1α is mainly expressed in the nucleus, and observed under a high-power microscope, 3 visual fields are randomly selected from each tissue section, and then the expression level is judged comprehensively according to the percentage of positive cells and staining intensity: the total positive score is 0 (less than 1%), 1 (1% ~ 10%), 2 (11% ~ 50%), 3 (51% ~ 80%), and 4 (> 80%). The staining intensity scores were 0 (no staining), 1 (light yellow), 2 (dark yellow), and 3 (dark brown). The EC was defined as high expression group when HIF-1 α positive cells were more than 50% and staining intensity ≥ 2 points, otherwise they were defined as low expression group (**Figures 2**, **3**).

### Statistical analysis

Statistical analysis was performed using SPSS 27.0 software (Chicago, IL, USA) and MedCalc15.2.2 software (MedC Software, Ostend, Belgium). The inter-class correlation coefficient (ICC) was employed to assess the agreement among the measurements provided by three observers. ICC values of 0.40 and 0.75 were utilized as thresholds for categorizing the consistency levels as low, medium, and high. The mean of the measurements obtained from the three observers was utilized for further analysis. The Kolmogorov-Smirnov test was conducted to evaluate the normality of the measurement data. The data adhering to a normal distribution were presented as mean ± standard deviation and analyzed using an independent sample t-test for group comparisons. Data not conforming to a normal distribution were represented as median (25th percentile, 75th percentile) and analyzed using the Mann-Whitney U test. Categorical data were expressed as frequencies and percentages, and group comparisons were conducted using the Chi-square test or Fisher’s exact test. Receiver operating characteristic (ROC) curve analysis was utilized to assess the predictive value of statistically significant parameters and their combinations in predicting low and high HIF-1α expression in EC. Binary logistic regression was employed to determine the predictive value of EC HIF-1α expression status in conjunction with independent risk factors. The area under the curve (AUC) was compared using the Delong test. A P-value less than 0.05 was considered statistically significant.

## Result

### Patient characteristics

Of the 72 EC patients finally enrolled, 32 (44.44%) were in the high HIF-1α expression group, and 40 (55.56%) were in the low HIF-1α expression group. Based on the pathological analysis, there were no statistically significant differences observed between the two groups in terms of age, differentiation degree, menopausal state, FIGO stage, DMI, LVSI, LNM, and pathological type. However, a significant difference was found between the two groups in presence of irregular vaginal bleeding (*P*=0.040), as presented in **Table 1**.

### Agreement on imaging parameters among the three observers

The three observers had high consistency on measurements of the MTR_asym_, ADC, D, D*, f, MK, FA, and MD values with the ICCs higher than 0.75, as shown in **Table 3**.

**Table 3:** Inter-observer agreement on the measurement of imaging parameters.

Parameters	Number	Observer1	Observer2	Observer3	ICC
MTR_asym_ (%)	Low HIF-1α expression(n=40)	2.67 ± 0.90	2.77 ± 0.86	2.76 ± 0.86	0.923
	High HIF-1α expression(n=32)	3.19 ± 0.86	3.22 ± 0.87	3.17 ± 0.85	0.992
ADC (×10^-3^mm^2^/s)	Low HIF-1α expression(n=40)	0.678 ± 0.19	0.682 ± 0.19	0.684 ± 0.19	0.983
	High HIF-1α expression(n=32)	0.86 ± 0.28	0.87 ± 0.29	0.87 ± 0.30	0.997
D (×10^-3^mm^2^/s)	Low HIF-1α expression(n=40)	0.49 ± 0.13	0.49 ± 0.12	0.49 ± 0.12	0.968
	High HIF-1α expression(n=32)	0.70 ± 0.28	0.71 ± 0.28	0.71 ± 0.28	0.998
D* (×10^-2^mm^2^/s)	Low HIF-1α expression(n=40)	0.52 ± 0.29	0.53 ± 0.30	0.54 ± 0.29	0.991
	High HIF-1α expression(n=32)	2.45 ± 2.98	2.29 ± 2.87	2.26 ± 2.83	0.969
f (%)	Low HIF-1α expression(n=40)	0.40 ± 0.14	0.40 ± 0.16	0.40 ± 0.16	0.938
	High HIF-1α expression(n=32)	0.23 ± 0.18	0.24 ± 0.19	0.24 ± 0.17	0.991
MK	Low HIF-1α expression(n=40)	0.60 ± 0.11	0.60 ± 0.11	0.60 ± 0.10	0.998
	High HIF-1α expression(n=32)	0.70 ± 0.12	0.71 ± 0.12	0.71 ± 0.12	0.998
MD (μm^2^/ms)	Low HIF-1α expression(n=40)	0.96 ± 0.19	0.95 ± 0.19	0.95 ± 0.19	0.995
	High HIF-1α expression(n=32)	1.20 ± 0.51	1.21 ± 0.54	1.21 ± 0.51	0.997
FA	Low HIF-1α expression(n=40)	0.33 ± 0.12	0.34 ± 0.12	0.34 ± 0.12	0.998
	High HIF-1α expression(n=32)	0.33 ± 0.11	0.33 ± 0.11	0.33 ± 0.10	0.999

ICC, Intra-group correlation coefficient; MTRasym, asymmetric magnetization transfer rate; ADC,Apparent diffusion coefficient; D, pure diffusion coefficient; D*, pseudo diffusion coefficient; f, perfusion fraction; MK, mean kurtosis; MD, mean diffusivity; FA, fractional anisotropy.

### Comparison of imaging parameters between high and low HIF-1α expression groups

**Figure 4:**
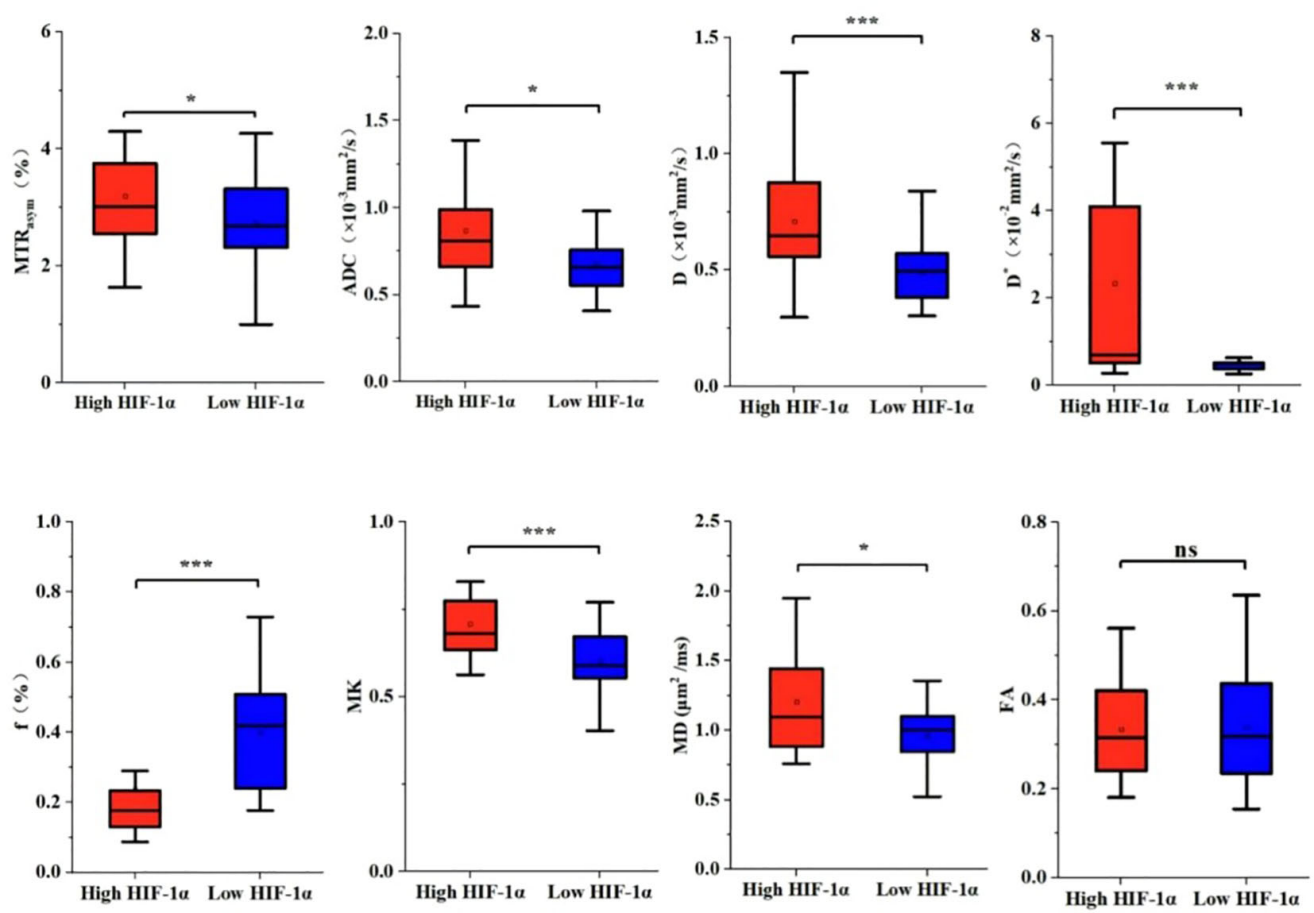
The histogram of EC parameters in HIF-1 α high expression group and HIF-1 α low expression group. The differences of MTRasym, ADC, D, D*, f, MK, MD and FA values between the two groups were compared. Note: *, *P* < 0.05; ***, *P* < 0.001.

The imaging parameters for high and low HIF-1α expression groups are presented in **Table 4**. MTR_asym_, ADC, D, D*, MK and MD values were significantly higher in high HIF-1α expression than those in low HIF-1α expression groups, whereas f value was significantly lower in high HIF-1α expression than in low HIF-1α expression groups (**Figure 4**).

**Table 4:** Comparison of imaging parameters between low HIF-1α expression and high HIF-1α expression patient groups.

Parameters	High HIF-1α expression n=32	Low HIF-1α expression n=40	t/z	*P*
**MTR_asym_ (%)**	3.19 ± 0.85	2.73 ± 0.85	2.126	***0.034***
**ADC (×10^-3^mm^2^/s)**	0.87 ± 0.29	0.68 ± 0.19	3.038	***0.002***
**D (×10^-3^mm^2^/s)**	0.71 ± 0.28	0.49 ± 0.12	3.810	***<0.001***
D* (×10^-2^mm^2^/s)	2.34 ± 2.81	0.53 ± 0.29	4.145	***<0.001***
**f (%)**	0.24 ± 0.18	0.40 ± 0.15	-4.757	***<0.001***
**MK**	0.708 ± 0.118	0.602 ± 0.108	3.621	***<0.001***
**MD (μm^2^/ms)**	1.207 ± 0.517	0.961 ± 0.190	1.989	***0.047***
**FA**	0.334 ± 0.107	0.339 ± 0.121	0.011	0.991

HIF-1α , Human epidermal growth factor receptor-2; MTRasym, Asymmetric magnetization transfer rate; ADC, Apparent diffusion coefficient; D, pure diffusion coefficient; D*, pseudo diffusion coefficient; f, perfusion fraction; MK, mean kurtosis; MD, mean diffusivity; FA, fractional anisotropy.

The bold values in the tables indicate p-values < 0.05, denoting statistical significance.

### Regression analyses

**Figure 5:**
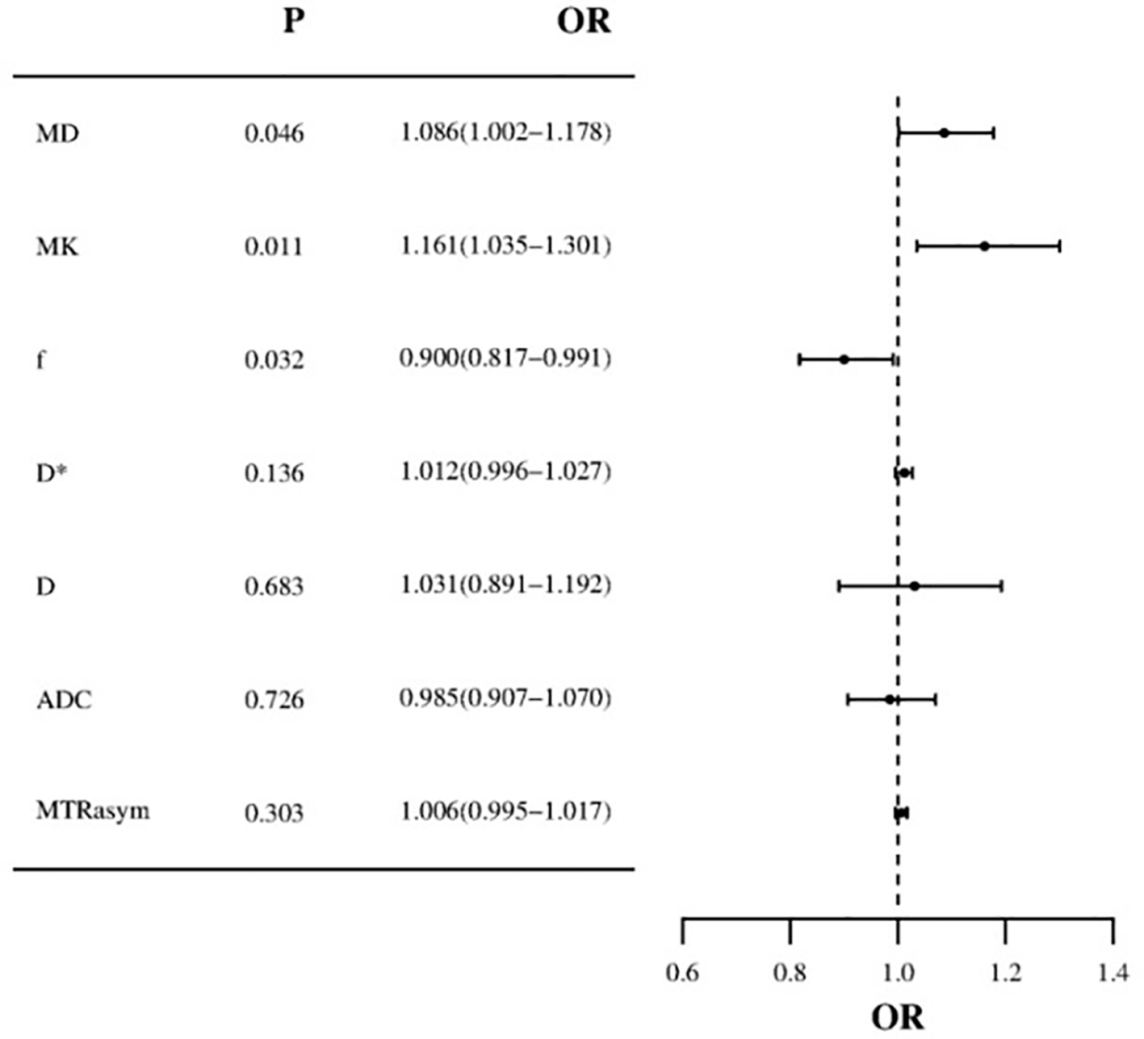
Forest plot of multivariate logistic regression (MD and MK values are risk factors for HIF-1α in EC, f value is protective factor for HIF-1α in EC).

Based on the comprehensive clinicopathological data and various quantitative parameters with *P* values below 0.1 in the comparison between the two groups, multiple linear regression analysis was conducted to assess covariance interference. It is found that except for D (11.368, excluded in the multiple linear regression analysis), the variance expansion factors (VIF) of other parameters were all less than 10 (MTR_asym_=1.104, ADC=6.654, D*=2.472, f=1.906, MK=1.135, and MD=1.251). Univariate analysis showed that irregular vaginal bleeding, MTR_asym_, ADC, D, D*, f, MK and MD were all helpful to evaluate the expression of HIF-1α in EC, but multivariate analysis showed that only f, MK and MD were independent predictors of HIF-1α expression in EC (**Table 5**, **Figure 5**).

**Table 5:** Univariate and multivariate analysis for identifying low HIF-1α expression and high HIF-1α expression patient groups.

Parameters	Univariate Analysis		Multivariate Analysis	
	OR (95%CI)	*P*	OR (95%CI)	*P*
Irregular vaginal bleeding	0.378(0.143 - 0.997)	***0.049***	0.545(0.053 - 5.594)	0.609
MTR_asym_	1.007(1.001 - 1.013)	***0.032***	1.006(0.995 - 1.017)	0.303
ADC	1.034(1.010 - 1.059)	***0.005***	0.985(0.907 - 1.070)	0.726
D	1.061(1.025 - 1.098)	***0.001***	1.031(0.891 - 1.192)	0.683
D*	1.015(1.002 - 1.027)	***0.026***	1.012(0.996 - 1.027)	0.136
f	0.939(0.907 - 0.973)	***<0.001***	0.900(0.817 - 0.991)	***0.032***
MK	1.102(1.039 - 1.169)	***0.001***	1.161(1.035 - 1.301)	***0.011***
MD	1.028(1.006 - 1.050)	***0.011***	1.086(1.002 - 1.178)	***0.046***

The bold values in the tables indicate p-values < 0.05, denoting statistical significance.

OR, odds ratio; CI, confidence interval; MTRasym, Asymmetric magnetization transfer rate; ADC,Apparent diffusion coefficient; D, pure diffusion coefficient; D*, pseudo diffusion coefficient; f, perfusion fraction; MK, mean kurtosis; MD, mean diffusivity; FA, fractional anisotropy.

### Ability of the imaging parameters to discriminate high HIF-1α expression from low HIF-1α expression groups

**Figure 6:**
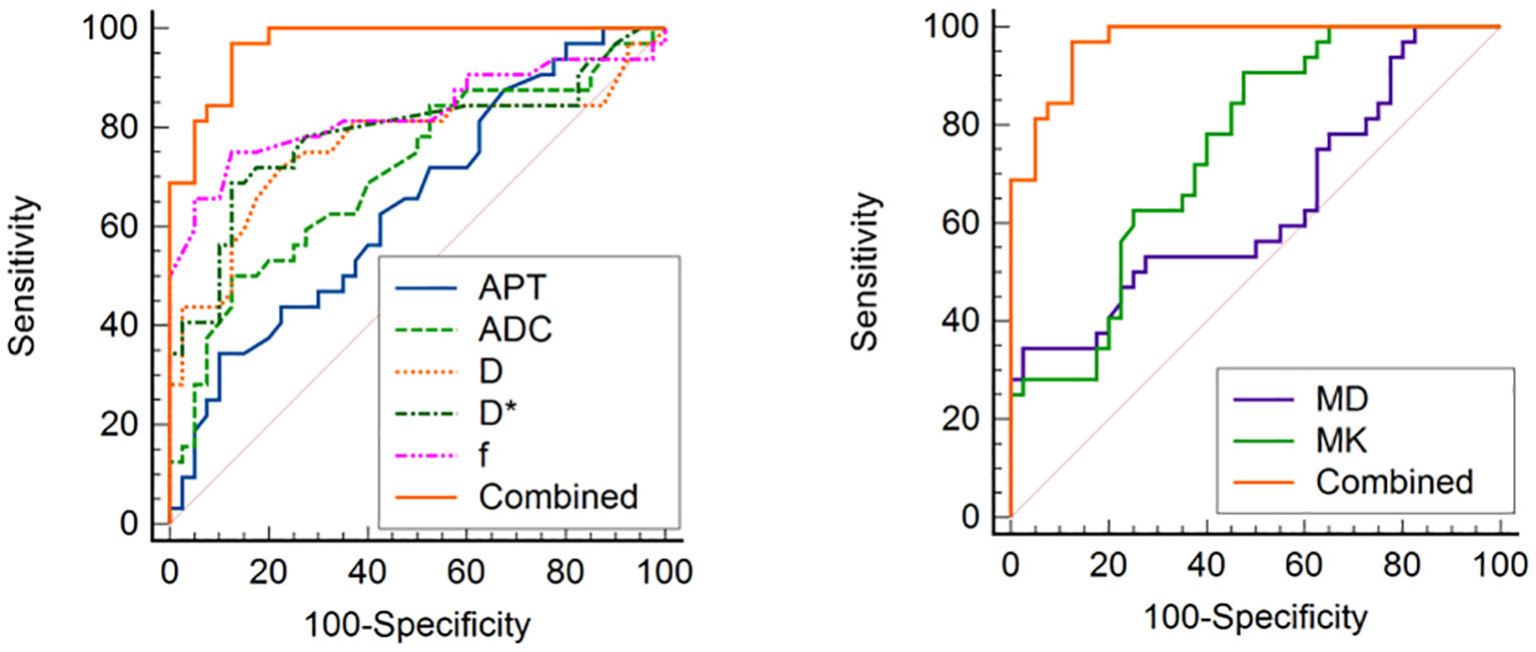
ROC curve analysis of the performance of each imaging parameter to evaluate the HIF-1α expression, AUCs of APT, ADC, D, D*, f, MD, MK, and Combined to evaluate the HIF-1α expression are 0.894 (0.740, 0.973), 0.746 (0.568, 0.879), 0.716 (0.528, 0.904), 0.920 (0.772, 0.984), 0.756 (0.578, 0.886), 0.973 (0.851–1.000), respectively.

ROC curves for APTw and multiple model DWI parameters and their combinations to discriminate high HIF-1α expression from low HIF-1α expression are shown in **Figure 6**. MTR_asym_, ADC, D, D^*^and f values commonly had good specificity (90.00%, 87.50%, 77.50%, 87.50% and 75.00%), while moderate sensitivity (65.60%, 50.00%, 71.90%, 68.80% and 87.50%). Combination of the above parameters showed significantly improved diagnostic performance with excellent sensitivity (96.90%) and specificity (85.00%) (**Table 6**). The ROCs for each parameters and their combination are shown in **Figure 6**.

**Table 6:** Predictive performance for identifying low HIF-1α expression and high HIF-1α expression EC.

Parameters	AUC (95% CI)	*P*	Cutoff	Sensitivity (%)	Specificity (%)	DeLong test	
MTR_asym_	0.646 (0.519 – 0.774)	***0.034***	3.515	65.60	90.00	Z =4.940	***<0.001***
ADC	0.709 (0.586 – 0.832)	***0.002***	0.815	50.00	87.50	Z=4.161	***<0.001***
D	0.763 (0.640 – 0.885)	***<0.001***	0.575	71.90	77.50	Z=3.335	***<0.001***
D*	0.784 (0.669 – 0.900)	***<0.001***	0.585	68.80	87.50	Z=3.300	***0.001***
f	0.828 (0.720 – 0.935)	***<0.001***	0.225	87.50	75.00	Z=2.608	***0.009***
MK	0.750 (0.639 - 0.860)	***<0.001***	0.593	90.60	52.50	Z=4.033	***<0.001***
MD	0.637 (0.505 - 0.769)	***0.047***	1.227	44.50	97.50	Z=4.976	***<0.001***
Combined (a.u.)	0.970 (0.938 - 1.000)	***<0.001***	0.251	96.90	85.00	NA	NA

MTRasym, asymmetric magnetization transfer rater; ADC, apparent diffusion coefficient; D, true diffusion; D*, pseudo diffusion; f, perfusion fraction; MK, mean kurtosis; MD, mean diffusivity; OR, odds ratio; CI, confidence interval; a.u., arbitrary unit; NA, not available.

The bold values in the tables indicate p-values < 0.05, denoting statistical significance.

### Correlation analysis between independent factors and HIF-1α expression

Spearman rank correlation analysis showed that there was an inverse correlation between f and HIF-1α expression level (rho = -0.565; *P* < 0.001). MK and MD showed positive correlations with the HIF-1α expression level (rho = 0.430, 0.316; both *P* < 0.001).

## Discussion

The major finding of this work was that multimodal quantitative MRI parameters, by APTw, DWI, DKI and IVIM, can be used to assess the HIF-1α expression in EC. We found that APT, ADC, D, D*, MK and MD values were significantly higher in high HIF-1α expression than in low HIF-1α expression groups, whereas f value was significantly lower in high HIF-1α expression than in low HIF-1α expression groups, meanwhile, the f value, MK value, and MD value are independent risk factors for predicting HIF-1α expression in EC. There was an inverse correlation between f and HIF-1α expression level, and there were positive correlations of MK value, and MD value with the HIF-1α expression level. The combination of different imaging parameters showed a significantly improved diagnostic efficacy in differentiation of HIF-1α expression in EC.

APTw imaging can be used to evaluate changes of intracellular protein concentration and tissue pH value with advantages of non-invasive and quantitative analysis (38–40). In this study, the MTR_asym_ of HIF-1 α high expression group was higher than that of low expression group, and the difference was statistically significant. The reason may be that the expression of HIF-1 α can regulate the metabolism and proliferation of local tumor (15), the number of cells in the local tumor increases and the metabolism is exuberant, which leads to the increase of local mobile proteins or peptides and the increase of MTR_asym_ in the group with high expression of HIF-1 α. Nuclear atypia, which induce the interaction between macromolecules and hydrophobic cell membrane and promote the release of proteins and peptides, may be another factor in the increase of MTR_asym_ in malignant tumors (41, 42). Although there was no difference in the pathological indexes of the degree of tumor differentiation, depth of myometrial invasion and tumor stage between the two groups (due to the small sample size and bias), previous studies (43, 44) showed that the tumors with higher expression of HIF-1α had lower tissue differentiation, deeper myometrial invasion, higher probability of lymph node metastasis and higher malignant degree of tumor. In addition, pH value is also one of the factors affecting MTR_asym_ (45, 46). Tumor with higher expression of HIF-1α can be associated with serious local hypoxia (47), where the tumor is mainly anaerobic metabolism, leading to increased production of local lactic acid and reduced pH value. However, the higher expression of HIF-1α can also induce the expression of vascular endothelial growth factor (VEGF) and other genes, which leads to the increase of tumor angiogenesis and local tumor microcirculation perfusion (48), and dilutes tumor local acidity to some extent. It may lead to the relative increase of pH value, which in turn leads to the increase of MTR_asym_ value. However, as mentioned above, the high expression of HIF-1α increases the expression of VEGF and other genes (49), tumor neovascularization, local tumor microcirculation perfusion, and dilutes tumor local acidity to some extent. It may lead to the relative increase of pH value, which in turn leads to the increase of MTR_asym_ value. At the same time, the perfusion parameter D* of the group with high expression of HIF-1α was higher than that of the group with low expression of HIF-1α, which further indicated that the perfusion of local microcirculation was increased in the group with high expression of HIF-1α. Compared with the results by Li et al. (42), the MTR_asym_ and D* values of cervical squamous cell carcinoma in poorly differentiated group were higher than those in well differentiated group, which also verified the hypothesis of the relationship between the change of MTR_asym_ value caused by the change of pH value and the perfusion parameter D* value of IVIM microcirculation.

The parameter D from the double exponential IVIM model (50) reflect the diffusion movement of water molecules without microperfusion (51), D* represents the diffusion effect caused by blood perfusion and reflects the perfusion of microcirculation in capillaries (52), and f reflects the percentage of the volume of water molecules in blood vessels to the volume of water molecules in the whole voxel (35). DKI is based on non-Gaussian distribution, which truly reflects that the movement of water molecules in living tissues is limited by tissue microstructure (53). The parameter MK by DKI can reflect the complexity of tissue structure, while MD value can reflect the overall diffusion level and diffusion resistance of water molecules (54). In this study, the parameters reflecting the diffusion of water molecules in EC (ADC, D and MD) were higher in the high HIF-1α expression than in the low expression group, which may be due to that the higher expression of HIF-1α may results in the increased proliferation of local tumor cells (15), and thus reduced extracellular space in EC. In addition, the MK value of the parameter reflecting the complexity of tumor tissue was also higher in the high HIF-1α expression than that in the low expression groups, which was related to the fact that the expression of HIF-1α could regulate the proliferation and epithelial mesenchymal transition of tumor cells, resulting in the exuberant proliferation of tumor local cells, the increase of epithelial stromal transition and the complexity of tumor local structure (15, 16). At the same time (55), the expression of HIF-1α can also regulate the apoptosis of tumor cells, which complicates the structural components of tumor tissue, which is another reason for the increase of MK in EC patients with high expression of HIF-1α. The D* and f values were higher in the group with high expression of HIF-1α, because the expression of HIF-1α could induce the expression of VEGF and other genes (49), which increased tumor angiogenesis and local microcirculation perfusion, which was consistent with the results of previous studies on IVIM to evaluate the expression level of HIF-1α in cervical cancer (35). However, in this study, the f value of EC in the high expression group of HIF-1α was lower than that in the low expression group EC. The reason may be that the value of f is not only related to microvessel density and blood flow velocity, but can also be related to the overall motion state of water molecules in tissue, vascular wall pressure and the b value setting in IVIM scan (51, 56–58). Pang et al. (59) found that when the b value varies from 0 to 700 s/mm^2^, the f value increases, while when the b value exceeds 700 s/mm^2^, the f value decreases. In this study, the b value of IVIM is between 0 and 2000 s/mm^2^, which makes the change trend of f value decrease with the increase of b. The high expression of HIF-1α associated with increased proliferation of tumor cells can results in the higher pressure on tumor neovascularization wall, which may slow down the blood flow velocity of local tumor microcirculation and lead to the reduced f value. Multivariate analysis showed that f value was a protective factor for the high expression of EC HIF-1α, and there was a negative correlation between f value and HIF-1α expression, which further indicated that the higher the f value, the weaker the invasive biological behavior of tumor cells and the lower the malignant degree of tumor, so the lower expression level of HIF-1α.

The shortcomings of this study are as follows: firstly, the sample size of this study is small, which needs to be further studied by increasing the sample size; secondly, the quantitative parameter measurement of this study avoids bleeding and necrosis, and does not outline the tumor globally, and some heterogeneity information may be omitted, which need to be studied in the future, such as texture analysis. The image quality of APTw, IVIM and DKI maps can be affected by respiratory movement, which needs to be optimized by respiratory trigger.

In summary, the quantitative parameters based on APTw and multi-model diffusion imaging can effectively evaluate the expression of EC HIF-1α, which has a certain prospect of clinical applications.

## Data Availability

The datasets presented in this article are not readily available because it contains sensitive information that cannot be shared publicly due to privacy and legal restrictions. Additionally, the datasets are part of an ongoing study, and making them publicly accessible at this stage could compromise the integrity of the research. We are willing to consider reasonable requests for access on a case-by-case basis, subject to appropriate approvals and agreements. Requests to access the datasets should be directed to JL, junjun54586@163.com.
