# Correction: Amide proton transfer-weighted imaging combined with multiple models diffusion-weighted imaging of endometrial cancer: correlations between multi-modal MRI parameters and HIF-1α expression

**DOI:** 10.3389/fonc.2025.1675936

**Published:** 2025-09-26

**Authors:** Jun Li, Changjun Ma, Shifeng Tian, Ailian Liu, Qingling Song, Nan Wang, Qingwei Song, Liangjie Lin, Peng Sun, Jiazheng Wang

**Affiliations:** ^1^ Department of Radiology, The First Affiliated Hospital of Dalian Medical University, Dalian, Liaoning, China; ^2^ School of Biomedical Engineering, Faculty of Medicine, Dalian University of Technology, Dalian, Liaoning, China; ^3^ Dalian Medical Image Artificial Intelligence Engineering Technology Research Center, Dalian, Liaoning, China; ^4^ Technology Innovation Center of Hyperpolarized MRI, Dalian, Liaoning, China; ^5^ Philips Health Technology (China) Co., Ltd., Beijing, China

**Keywords:** DWI, IVIM, HIF-1α, endometrial cancer, amide proton transfer weighted imaging

There was a mistake in **Figure 2** and **Figure 3** as published. Both of these figures contained unintended internal duplication. The corrected **Figure 2** and **Figure 3** appear below.

There was a mistake in the captions of **Figure 2** and **Figure 3** as published. The captions have been revised to correspond with the updated figures. The corrected captions of **Figure 2** and **Figure 3** appear below.

**Figure 2:**
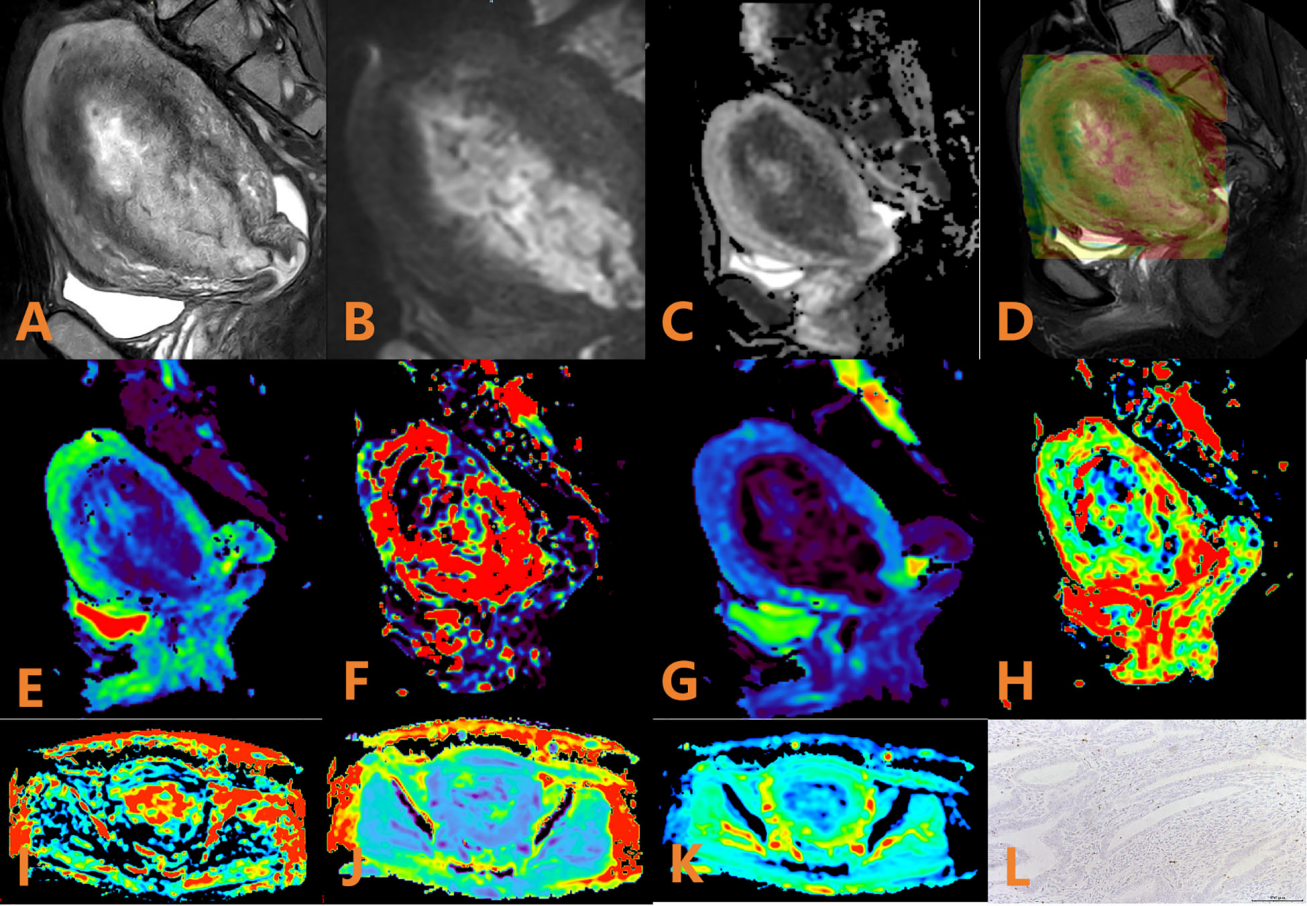
APTw, DKI and IVIM parameters for an EC patient with low HIF-1α expression. **(A)** sagittal T2WI, showing a slightly high signal mass in the uterine cavity; **(B)** sagittal DWI image; **(C)** sagittal ADC image; **(D)** APTw fused with T2WI (mean MTRasym value 3.53%); **(E–H)** ADC, D, D*, and f images. Mean values are 0.600 × 10–^3^ mm^2^/sec for ADC, 0.410 × 10–^3^ mm^2^/sec for D, 0.280 × 10–^2^ mm^2^/sec for D* and 0.57% for f; **(I–K)** FA, MK, and MD images. Mean values are 0.388 for FA, 0.586 for MK, and 1.147 μm^2^/ms for MD; **(L)** Immunohistochemical staining image (×200) showed that HIF-1α expression of the tumor appeared as low expression.

**Figure 3:**
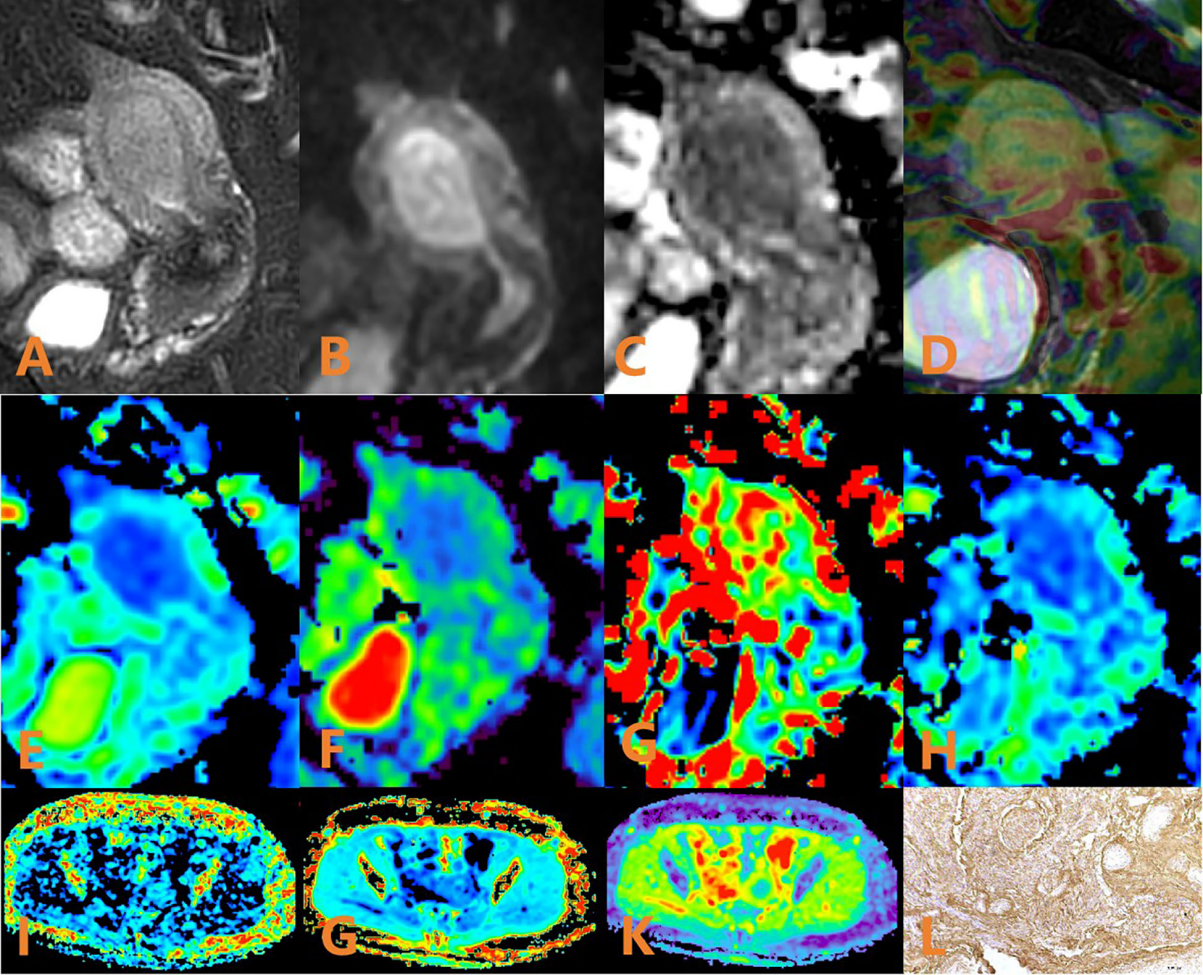
APTw, DKI and IVIM in EC that had high HIF-1α expression. **(A)** sagittal T2WI, showing a slightly high signal mass in the uterine cavity; **(B)** sagittal DWI image; **(C)** sagittal ADC images; **(D)** APTw and T2WI fusion images (mean APT value 1.63%) **(E–H)** ADC, D, D*, and f images. Mean values are 0.950×10–^3^ mm^2^/sec for ADC, 0.890 × 10–^3^ mm^2^/sec for D, 4.780 × 10–^2^ mm^2^/sec for D* and 0.130% for f; **(I–K)** FA, MK, and MD images. Mean values are 0275 for FA, 0.566 for MK, and 0.966μm^2^/ms for MD; **(L)** Immunohistochemical staining image (×200) showed that HIF-1α expression of the tumor appeared as high expression.

